# Identification of *mcr-2* and *mcr-3* Genes in Colistin-Resistant *E. coli* O157:H7 Isolated From Raw Meat Samples in Beirut, Lebanon

**DOI:** 10.1155/ijm/8079270

**Published:** 2025-02-27

**Authors:** Fatima H. Fneish, Souraya A. Domiati, Khaled H. Abd El Galil

**Affiliations:** ^1^Department of Pharmaceutical Sciences, Faculty of Pharmacy, Beirut Arab University, Beirut, Lebanon; ^2^Department of Pharmacology and Therapeutics, Faculty of Pharmacy, Beirut Arab University, Beirut, Lebanon; ^3^Department of Microbiology and Immunology, Faculty of Pharmacy, Mansoura University, Mansoura, Egypt

## Abstract

Colistin is a last-resort antibiotic used to treat multidrug-resistant Gram-negative bacterial infections. The global emergence of colistin resistance has been attributed to plasmid-mediated mobile colistin resistance (*mcr*) genes. In Lebanon, bacteria carrying the *mcr-1* gene have increasingly been identified in food animal sources. This study is aimed at detecting colistin-resistant Shiga toxigenic *Escherichia coli* O157:H7 in raw meat samples from local markets in the suburbs of Beirut and evaluating their antimicrobial resistance profiles.

A total of 50 meat samples, including 25 minced beef and 25 burger samples, were collected and analyzed. Antimicrobial resistance patterns were determined using the Kirby–Bauer method, while colistin resistance and the presence of *mcr-2* and *mcr-3* genes were assessed using broth microdilution and PCR amplification techniques. Among these samples, 23 (46%) tested positive for *E. coli* O157:H7. Resistance to ampicillin and amoxicillin/clavulanic acid was observed in 96% of the samples, while 61% were resistant to trimethoprim/sulfamethoxazole, and 43% to chloramphenicol. Notably, 87% of the samples displayed colistin resistance, with a minimum inhibitory concentration (MIC) of ≥ 4 *μ*g/mL. The *mcr-2* gene was present in four isolates (17.4%), and the *mcr-3* gene was identified in 10 isolates (43.4%).

This study is the first to document the presence of plasmid-mediated colistin resistance genes, *mcr-2* and *mcr-3*, in *E. coli* O157:H7 strains in Lebanon. These findings highlight a serious public health concern for the Lebanese community. Therefore, the responsible use of antibiotics across all healthcare sectors, combined with strict hygiene measures in food handling, is essential to control the spread of colistin-resistant genes.

## 1. Introduction

Shiga toxin (STX)-producing *Escherichia coli* (STEC) is a significant foodborne pathogen responsible for causing a range of diseases such as watery diarrhea, hemorrhagic colitis (HC), and hemolytic uremic syndrome (HUS) [1]. *STEC* O157:H7 was first documented in 1982, [2], and until now, it remains the most dominant serotype associated with outbreaks worldwide [3]. Ruminants and cattle are recognized as natural reservoirs for this bacterium that can be transmitted to humans by direct contact with infected animals or by the ingestion of contaminated foods and water [4]. Treatment of infections caused by *E*. *coli* O157:H7 using antibiotics is debatable. Many studies have shown that the use of antibacterial agents is associated with an increased risk of HUS due to stimulation of STX release [5–8]. In contrast, Percivalle et al. demonstrated that colistin inhibits STX release, suggesting it as a good option for treating *E. coli* O157 H7 infections [9].

Colistin (Polymixen E) is a polypeptide antibacterial agent extracted from the soil bacterium *Paenibacillus polymyxa* var. *colistinus* that has been used clinically after the FDA approval in the 1950s [10]. This antibiotic is characterized by its bactericidal effect against Gram-negative bacteria and most members of the family *Enterobacteriaceae* [11, 12]. Over the years, the use of colistin in humans has decreased due to its significant toxicity and is now limited to some cases of cystic fibrosis [13, 14]; in contrast, its use in the veterinary field has increased to treat intestinal infections and as a growth promoter in farmed animals [15–17]. Recently, with the emergence of multidrug-resistant (MDR) bacteria, the use of colistin in clinical practice has resumed as the last resort antibiotic [18–20]. The World Health Organization (WHO) has classified colistin as a crucial drug of “very high importance for human medicine” [21].

Unfortunately, the extensive use of this antibiotic, particularly in the animal sector, has led to a global increase in colistin-resistant pathogens [22–25]. This issue became a major concern after discovering the first plasmid-mediated mobile colistin resistance gene (*mcr-1*) in China in 2015, indicating that colistin resistance can spread through horizontal transfer [26]. Since then, several variants of *mcr* genes (*mcr*-2 to *mcr*-10) have been revealed globally [27–37]. Bacteria harboring these genes have been isolated from animals, humans, food products, and many environmental settings such as hospital manure, rivers, and seas [38–41]. It is largely recognized that the dissemination of *mcr* genes between humans and animals primarily occurs through the food chain and environmental pollution [42]. These findings constitute an alarming threat as they can compromise the efficacy of colistin in treating infections caused by MDR pathogens [43].

In Lebanon, the detection of *mcr* variants in various bacterial species highlights the increasing concern over colistin resistance. *E. coli* carrying the *mcr-1* gene has been detected in environmental water samples [44], swine [45], and poultry [46], while *mcr-8.1* has been found in *Klebsiella pneumoniae* and *mcr-9* in *Enterobacter hormaechei* [47, 48]. Despite these findings, there is a lack of comprehensive data on the prevalence of colistin-resistant bacteria in meat products. Furthermore, the presence and distribution of other *mcr* variants, such as *mcr-2* and *mcr-3*, have not been studied in Lebanon, resulting in significant gaps in understanding the full scope of colistin resistance in the region.

This study is aimed at assessing the prevalence of colistin resistance in *E. coli* O157:H7 strains isolated from raw meat samples in Beirut, Lebanon, with a particular focus on the *mcr-2* and *mcr-3* variants.

## 2. Materials and Methods

### 2.1. Isolation of *E. coli* O157:H7 From Meat Samples

Fifty raw meat samples (25 minced beef and 25 burger samples) were randomly collected from various butcheries located in the suburbs of Beirut, Lebanon, including Basta, Jnah, Chiyah, Haret Hreik, and Borj El Barajneh. Samples were examined within 2 h of collection and transported to the laboratory under cold conditions. The study was conducted at the microbiology lab at Beirut Arab University. Detection of *E. coli* O157:H7 in meat samples was performed using the procedure previously described by Hessain et al. [49]. Concisely, 25 g of each sample was added into 225 mL of tryptone soya broth containing 20 mg/L novobiocin and incubated at 37°C for 16–18 h. The enriched cultures were then subcultured into eosin methylene blue (EMB) agar media and incubated at 37°C for 24 h. The distinctive green metallic shine colonies of *E. coli* were further cultured on tellurite (2.5 mg/L)–cefixime (0.05 mg/L)–sorbitol–MacConkey (TC-SMAC) agar and incubated at 37°C for 24 h [49].

### 2.2. Serological Testing of *E. coli* O157:H7

The colonies that did not ferment sorbitol on the TC-SMAC agar were subjected to a slide agglutination test using an *E. coli* O157 latex kit (Liofilchem product). If agglutination occurred within 1 min, these colonies were classified as *E. coli* O157 positive.

### 2.3. Molecular Identification of *E. coli* O157:H7

Polymerase chain reaction (PCR) was performed using *E. coli* O157:H7 specific primers (Table 1) to detect the presence of *rfbE* (somatic antigen O157) and *fliC* genes (flagellar antigen). PCR amplification was done in a total volume of 20 *μ*L containing 5 *μ*L total DNA, 10 *μ*L 5xFIREPol master mix, 1 *μ*L from each primer, and up to 20 *μ*L with nuclease-free water. The PCR cycling conditions were set to be 95°C for 5 min as the predenaturation step followed by 35 cycles, each consisting of denaturation at 94°C for 30 s, annealing at 60°C for 30 s (*rfbE*-O157 gene) and 68°C for 30 s (*fliC* gene), and extension at 72°C for 50 s with a final extension at 72°C for 5 min. The amplification products were then analyzed using agarose gel electrophoresis with a 100 bp DNA ladder (Invitrogen).

### 2.4. Evaluation of Antibacterial Susceptibility Using Disc Diffusion Method

The Kirby–Bauer diffusion method was used to determine the susceptibility of the isolated strains to 15 different antibacterial agents as shown in Table 2 [52]. Bacterial inocula (25 *μ*L), equivalent to a 0.5 McFarland standard, were swabbed over the surface of the solidified Mueller–Hinton agar (MHA) plates and dried for 10 min. Antibiotic discs were distributed on MHA plates using an Oxoid antibiotic dispenser. The plates were left at room temperature for 15 min to allow prediffusion of the chosen antibacterial agents. They were then incubated at 37°C for 18–24 h [52]. Zones of inhibition were measured with a vernier caliper to the nearest millimeter. Data were categorized as susceptible (S), intermediate (I), or resistant (R) based on the standards of inhibition zone diameters defined by the Clinical and Laboratory Standards Institute guidelines [53].

### 2.5. Determination of Minimum Inhibitory Concentration (MIC) of Colistin Among the Collected Isolates

The broth microdilution method was used to determine the MICs for colistin among *E. coli* O157:H7 isolates following the EUCAST/CLSI guidelines. Various colistin dilutions ranging from 0.25 to 128 *μ*g/mL were prepared and inoculated with *E. coli* O157: H7 samples to achieve a final concentration of 5 × 10^5^ CFU/mL in each well. This method was performed in triplicate. The microtiter plates containing the bacterial cultures were incubated at 37°C for 24 h and then examined for microbial growth. MIC values were determined as the lowest concentration of colistin that inhibited bacterial growth. The breakpoint for the analysis of MIC against colistin was set as stated by CLSI 2021. An MIC ≤ 2 *μ*g/mL was considered as intermediate while an MIC ≥ 4 *μ*g/mL was classified as resistant [53, 54].

### 2.6. Molecular Detection of Colistin Resistance Genes Among *E. coli* O157:H7 Isolates

PCR was performed to detect the presence of *mcr*-2 and *mcr*-3 genes in colistin-resistant isolates using specific primers [35, 37]. The sequences of the primers are provided in Table 3. The amplification process was carried out according to the following program: initial predenaturation at 95°C for 5 min, 35 cycles of denaturation at 94°C for 30 s, and then annealing for *mcr*-2 at 55°C and for *mcr*-3 at 58°C for 30 s, an extension at 72°C for 30 s, and a final extension at 72°C for 5 min. At the end of the process, the amplification products were analyzed using agarose gel electrophoresis and compared with a 100 bp DNA ladder.

## 3. Results

### 3.1. Prevalence of *E*. *coli* O157:H7 Among Meat Samples

**Figure 1:**
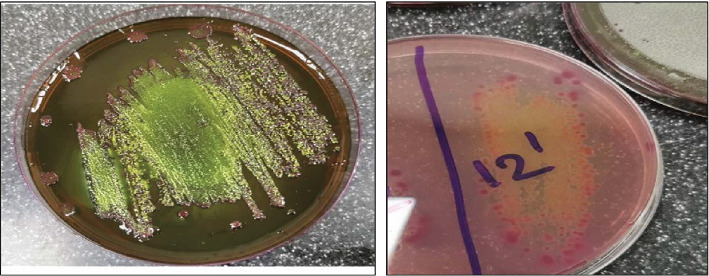
Growth of some of the collected *E. coli* isolates on EMB agar and TC-SMAC agar.

**Figure 2:**
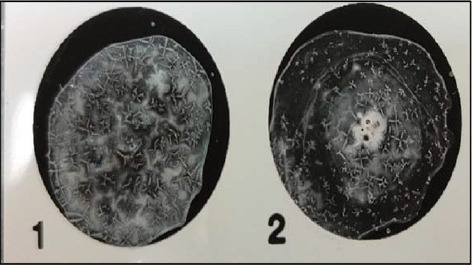
Positive latex agglutination test of isolate Nos. 12 and 15.

**Figure 3:**
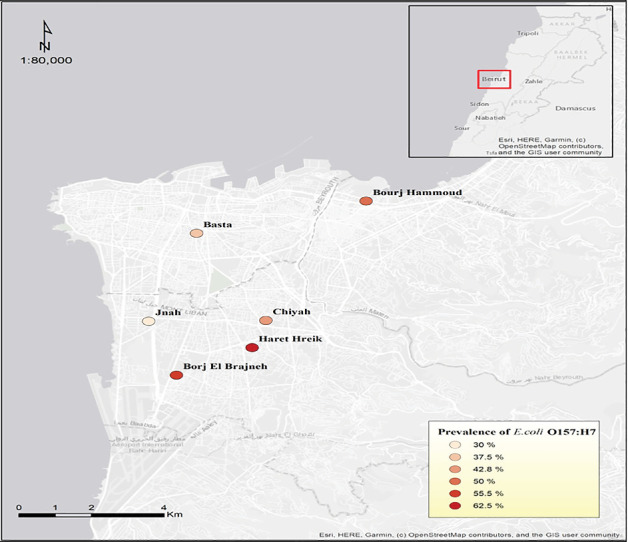
Study area map: prevalence of *E. coli* O157:H7 in meat samples (minced+burger) from Beirut suburbs.

All suspected *E. coli* samples isolated from meat samples that grew on EMB-producing metallic green sheen colonies were selected and differentiated on TC-SMAC agar. Colorless, nonlactose fermenting colonies indicative of the existence of *E. coli* O157:H7 were further tested using the latex agglutination test (Figures 1 and 2). Of the 50 raw meat samples examined, 23 (46%) tested positive for *E. coli* O157:H7 serotypes. Specifically, *E. coli* O157:H7 was detected in 12 (48%) of minced beef samples and 11 (44%) of burgers. Prevalence rates across different suburbs demonstrated some variability, with lower rates in Jnah (30%) and Basta (37.5%), moderate rates in Chiyah (42.8%) and Borj Hammoud (50%), and higher rates in Borj El Brajneh (55.5%) and Haret Hreik (62.5%). These findings are illustrated in Table 4 and Figure 3.

### 3.2. Molecular Identification of *E. coli* O157: H7

**Figure 4:**
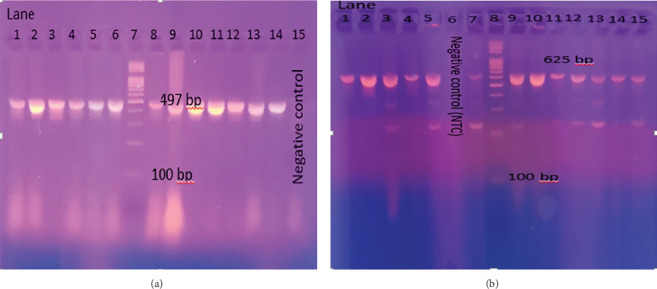
Agarose gel electrophoresis of DNA fragments generated by PCR for (a) *rfbE* gene and (b) *fliC* gene of *E. coli* O157:H7 isolates. (a) Lane 7: DNA molecular weight marker (100 bp); Lane 15: negative control; Lanes 1, 2, 3, 4, 5, 6, 8, 9, 10, 11, 12, 13, and 14: *E. coli* O:157:H7 isolates. (b) Lane 8: DNA molecular weight marker (100 bp); Lane 6: negative control; Lanes 1, 2, 3, 4, 5, 7, 9, 10, 11, 12, 13, 14, 15: *E. coli* O:157:H7 isolates.

The probable isolates of *E. coli* O157:H7 that gave a positive latex agglutination test underwent molecular confirmation using specific primers for the detection of *rfbE* and *fli*C genes. PCR products with an amplicon size of 497 bp were considered indicative of the existence of the *rfbE* gene. On the other hand, an amplicon size of 625 bp was indicative of the *fliC* gene as shown in Figure 4.

### 3.3. Antimicrobial Susceptibility Testing

*E. coli* O157: H7 isolates recovered from meat samples were screened for antibiotic susceptibility against the chosen 15 antibacterial agents. Nighty-six percent of all samples (minced and burger) were resistant to ampicillin and amoxicillin/clavulanic acid, 61% were resistant to trimethoprim/sulfamethoxazole, and 43% were resistant to chloramphenicol. The pattern of resistance to these antibiotics was higher in burger samples compared to minced beef samples. Lower rates of resistance were seen against piperacillin, ciprofloxacin, ceftriaxone, ceftazidime, and gentamicin. Nevertheless, high sensitivity rates (> 90%) were detected against meropenem, cefepime, aztreonam, and piperacillin/tazobactam as shown in Table 5.

### 3.4. Broth Microdilution Testing Against Colistin

The values of MICs for colistin ranged from 1 to 128 *μ*g/mL. Twenty isolates of *E. coli* O157:H7 (beef and burger samples) showed resistance to colistin (87%), whereas two isolates were considered to be colistin intermediate and only one isolate showed susceptibility to colistin according to the MIC breakpoints of colistin [53] (Table 6).

### 3.5. Molecular Detection of mcr-2 and mcr-3 Genes

**Figure 5:**
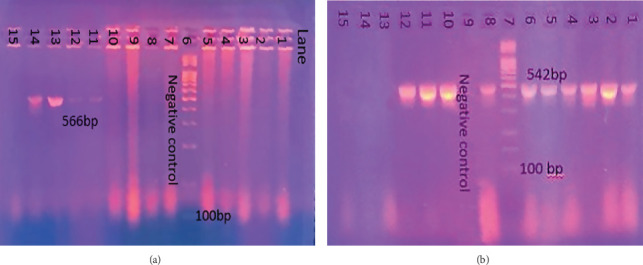
Agarose gel electrophoresis of DNA fragments generated by PCR for *mcr-2* gene (Figure A) & *mcr-3* gene (Figure B) of *E. coli* O157:H7 isolates. (a) Lane 6: DNA molecular weight marker (100 bp); Lane 7: negative control; Lanes 11, 12, 13, and 14: *E. coli* O157:H7 samples carrying *mcr-2* gene. (b) Lane 7: DNA molecular weight marker (100 bp); Lane 9: negative control; Lanes 1, 2, 3, 4, 5, 6, 8, 10, 11, and 12: *E. coli* O157:H7 samples carrying *mcr-3* gene.

The total number of *E. coli* O157:H7 *isolates* was further examined for the existence of the *mcr-2* and *mcr-3* genes using PCR with specific primers. Accordingly, the *mcr-2* gene was identified in four isolates (17.4%), while the *mcr-3* gene was spotted in 10 isolates (43.4%) as seen in Figure 5 and Table 6. The prevalence of *mcr-3* genes in *E. coli* O157:H7 obtained from minced beef was higher than that found in burger samples with a percentage of 58.3% and 27.2%, respectively. Only one isolate (a minced beef isolate) was found to harbor both genes at the same time (Sample 20). All isolates carrying *mcr*-2 and *mcr*-3 genes were phenotypically resistant to colistin based on microdilution test results. However, some of the isolates showed contradictory features such as (Sample 27) which carried some of the *mcr* genes but did not demonstrate resistance to colistin or showed resistance to colistin without harboring any of the tested *mcr* genes (Samples 1, 12, 22, 23, 26, 28, 29 & 32).

The distribution of *mcr-2* and *mcr-3* genes varied across the sampled areas in Lebanon. In Chiyah, neither gene was detected, indicating the absence of these resistance markers. In Haret Hrek, the *mcr-3* gene was identified, while *mcr-2* was not observed. Conversely, in Basta, only the *mcr-2* gene was detected, with no evidence of *mcr-3*. In Borj Hammoud, the *mcr-3* gene was present, but *mcr-2* was not found. In Borj al Barajneh, both *mcr-2* and *mcr-3* genes were detected, and similarly, in Jnah, both genes were identified. These findings reveal a diverse distribution of *mcr-2* and *mcr-3* across the sampled regions, with some areas exhibiting the presence of one or both genes, while others were completely free of these resistance markers.

## 4. Discussion

Contaminated meat and its products are considered a major cause of *E. coli* O157:H7 infections in humans. Animals such as cattle, sheep, and goats play a significant role in spreading this pathogen [55, 56]. Our data showed that *E. coli* O157:H7 was present in 46% of raw meat samples. This result was similar to that obtained in a study conducted in 2018 in Ghoubairy, a suburban region of Beirut, in which the prevalence of *E. coli* O157:H7 was 43%. Lower proportions were detected in different Lebanese areas such as Sin el Fil (21%), Al Hadath (12%), and Antelias (11%) [57]. Moreover, our data revealed that the existence of this pathogen in minced beef collected from butcheries located in the Beirut region was higher than that observed in Tripoli, a region located north of Beirut (18.8%) [58]. In contrast, reports from other countries documented lower prevalence in meat products [59–66]. The contamination of meat by *E. coli* O157:H7 typically occurs throughout the slaughtering process. Equipment and tools used in the slaughterhouse contribute to the transmission of this microorganism to meat products [67]. Consequently, the high rates of contamination by *E. coli* O157:H7 observed in this study may indicate that meat is prepared and produced under poor hygienic conditions.

Antimicrobial resistance often referred to as the silent pandemic is regarded today as a global public health issue where urgent actions are needed to prevent treatment failure of many human infections [68]. According to the antimicrobial susceptibility patterns, all *E. coli* O157:H7 isolates were classified as MDR pathogens with resistance to at least three antibiotics from different classes. Similar findings on multidrug resistance have been reported from other parts of the world [69, 70].

Isolates of *E. coli* O157:H7, in this study, exhibited very high resistance to amoxicillin which was consistent with Gugsa et al. (100%), Tadese et al. (100%), and Abdissa et al. (100%) [71–73]. Additionally, 96% of the isolates were resistant to amoxicillin/clavulanic acid. This result aligns with a previous study conducted in Egypt that reported a complete resistance (100%) of *E. coli* O157:H7 isolated from food products of animal origin against this antibiotic [70]. In contrast, some reports demonstrated a complete susceptibility of *E. coli* O157:H7 to amoxicillin/clavulanic acid [74–77]. Resistance to chloramphenicol in this research was recorded in 41% of isolates. Likewise, a high prevalence of resistance against chloramphenicol in the *E. coli* O157:H7 strains was reported in Nigeria [78] and Iran [79, 80]. Furthermore, our data revealed a high level of resistance against trimethoprim/sulfamethoxazol (61%) which was inconsistent with many reports declaring complete sensitivity to this antibacterial agent by *E. coli* O157:H7 isolates [70, 81–83]. In this study, cephalosporins demonstrated good activity against our isolates with a sensitivity rate of 78%, 91.3%, and 96% for ceftriaxone, ceftazidime, and cefepime, respectively. The collected isolates were also susceptible to imipenem (61%), gentamicin (65.2%), ciprofloxacin (69.5%), and norfloxacin (91.3%). Comparable results regarding the efficacy of these antibacterial agents against *E. coli* O157:H7 were also confirmed by several investigations [84–90]. None of the isolates were resistant to aztreonam which is in agreement with findings reported by other investigators [77, 91, 92]. The high rate of resistance observed in the present study can be attributed to the inappropriate and extensive use of antibiotics for therapeutic and prophylactic purposes in the veterinary sector. According to Dankar et al., the most commonly used antimicrobials by dairy farmers in Lebanon were penicillins, amoxicillin, and sulfonamides [93]. Consequently, the resistance of *E. coli* O157:H7 isolates in our study to amoxicillin, amoxicillin/clavulanic acid, and trimethoprim/sulfamethoxazole is to be expected.

Colistin resistance was detected in 87% of *E. coli* O157:H7 isolates based on the results of the broth microdilution test (MIC ≥ 4 *μ*g/mL). The prevalence of *mcr-2* and *mcr-3* genes among the isolates under study was 17.4% and 43.4%, respectively. In a previous study, Xavier et al. [35] reported the first appearance of the *mcr*-2 gene in Belgium. Later, Yin et al. [37] discovered the presence of the *mcr*-3 gene in *E. coli* of pig origin. Since the first discovery of the plasmid mediated-colistin resistance gene (*mcr-1)* in China, several studies have been performed to detect the occurrence of variants of *mcr* genes in *E. coli* isolates from different sources [94–100]. In China, several studies have been conducted to detect the occurrence of *mcr* gene variants in *E. coli* isolates from various sources [94–100]. However, there has been insufficient research focusing on colistin resistance and the presence of *mcr* genes in STX-producing *E. coli* O157:H7. In the United States, the prevalence of *mcr-1* and *mcr-2* genes in STX-producing *E. coli* isolates (O157 and non-O157) recovered from livestock and water samples was assessed by Mavrici et al. [101]. Unlike our findings, *mcr* genes were not detected in their study. In Spain, *STEC* isolates were found to harbor *mcr-1*, *mcr-4*, and *mcr-5* genes [102]. Similarly, in China, the *mcr-1* gene was detected in *STEC*, including O157 serogroups resistant to colistin [103, 104]. In line with our results, Ayaz et al. reported the detection of *mcr-2* and *mcr-3* genes in *E. coli* O157:H7 isolates, although at a lower prevalence rate (10.2%) [105]. The cooccurrence of *mcr* genes has been observed in several studies [95, 102, 106]. Notably, we also detected the coexistence of *mcr-2* and *mcr-3* genes in one *E. coli* O157:H7 isolate, a finding that has been reported by others [105].

Colistin resistance can be mediated by many approaches: chromosomal mutation in genes associated with the primary target of colistin, efflux pump, and the horizontal transfer of *mcr* genes [107]. The existence of these different resistance mechanisms, combined with the presence of multiple *mcr* gene variants, helps explain why some strains in our study exhibited phenotypic colistin resistance despite not harboring *mcr* genes. These strains may be resistant through chromosomal mutations and efflux pump activity or by harboring *mcr* gene variants not explored in this study.

In some countries like the United States, Finland, and Norway, colistin is prohibited for use in food-producing animals [15]. In Lebanon, the Ministry of Agriculture banned the use of colistin in the animal sector in 2022 [108]. However, before the regulations implemented by the Lebanese government to restrict the use of colistin, the antibiotic was widely available for both human and veterinary use. According to data obtained from the Order of Pharmacists of Lebanon (OPL), the amount of colistin imported for hospital use increased five-fold from 2010 to 2017. The heavy uncontrolled use of colistin in Lebanon over the years before recent policy changes has played a role in the emergence and dissemination of *mcr*-mediated colistin resistance [109]. Studies have already reported the detection of colistin resistance in clinical settings as well as poultry farms and water sources [110–112]. The colistin-resistant bacteria identified include *E*. *coli*, *Klebsiella pneumonia*, and *Proteus mirabilis* isolates. Remarkably, the presence of the *mcr-1* gene has been reported in these bacterial isolates [113–116].

## 5. Conclusion

To our knowledge, this is the first study to report the occurrence of the *mcr-2* and *mcr-3* genes in *E. coli* O157:H7 isolated from meat in Lebanon as well as the Middle East region. This finding underscores the need for further investigations to assess the clinical and epidemiological significance of these resistance genes. However, given the serious public health risk posed by the emergence and spread of this *mcr*-mediated colistin-resistant food-borne pathogen, it is essential to take urgent multisectorial actions to enhance surveillance and implement a national strategy aimed at preventing further dissemination.

## Figures and Tables

**Table 1:** PCR primers for the molecular identification of *E. coli* O157:H7 isolates.

**Primers**	**Sequence 5**⁣′**-3**⁣′	**Amplicon size**	**Reference**
rfbE-O157 F	CATTGGCATCGTGTGGACAG	497 bp	Desmarchelier et al. [50]
rfbE-O157 R	AAGATTGCGCTGAAGCCTTTG		
fliC-H7 F	GCGCTGTCGAGTTCTATCGAGC	625 bp	Gannon et al. [51]
fliC-H7 R	CAACGGTGACTTTATCGCCATTCC		

**Table 2:** List of antibacterial agents used in *E. coli* O157:H7 antibacterial susceptibility testing.

**Antimicrobial agent**	**Symbol**	**Disc content (*μ*g/disc)**	**Zone diameter (mm)**		
			**S**	**I**	**R**
Piperacillin/tazobactam	TZP	110	≥ 21	18–20	≤ 17
Amoxicillin/clavulanic acid	AMC	30	≥ 18	14–17	≤ 13
Piperacillin	PRL	100	≥ 21	18–20	≤ 17
Ampicillin	AMP	10	≥ 17	14–16	≤ 13
Ceftriaxone	CRO	30	≥ 23	20–22	≤ 19
Ceftazidime	CAZ	30	≥ 21	18–20	≤ 17
Cefepime	FEP	30	≥ 25	19–24	≤ 18
Imipenem	IPM	10	≥ 23	20–22	≤ 19
Meropenem	MEM	10	≥ 23	20–22	≤ 19
Chloramphenicol	C	30	≥ 18	13–17	≤ 8
Gentamicin	CN	10	≥ 15	13–14	≤ 12
Ciprofloxacin	CIP	5	≥21	16–20	≤15
Norfloxacin	NOR	10	≥17	13–16	≤12
Aztreonam	ATM	30	≥21	18–20	≤17
Trimethoprim–sulfamethoxazole	SXT	1.25/23.75	≥16	11–15	≤10

**Table 3:** PCR primers for the detection of *mcr* genes in *E. coli* O157:H7 isolates.

**Primer**	**Sequence 5**⁣′**-3**⁣′	**Annealing temp**	**Amplicon size**	**Reference**
*mcr-2* F	TGTTGCTTGTGCCGATTGGA	55°C	566 bp	Xavier et al. [35]
*mcr-2* R	AGATGGTATTGTTGGTTGCG			
*mcr-3* F	TTGGCACTGTATTTTGCATTT	58°C	542 bp	Yin et al. [37]
*mcr-3* R	TTAACGAAATTGGCTGGAAA			

**Table 4:** Prevalence of *E. coli* O157:H7 in minced meat and burger samples across different suburbs of Beirut.

**Location**	**Minced** **N** = 25	**Burger** **N** = 25	**Positive minced** **N** = 12	**Positive burger** **N** = 11	**Total positive** **N** = 23	**Total (%)**
Chiyah	3	4	2	1	3	42.8%
Basta	4	4	1	2	3	37.5%
Haret Hreik	4	4	3	2	5	62.5%
Burj al Barajneh	5	4	2	3	5	55.5%
Bourj Hammoud	4	4	2	2	4	50.00%
Jnah	5	5	2	1	3	30%

*Note: N*: the total number of samples analyzed for each category (minced meat or burger). Positive minced: the number of minced meat samples testing positive for *E. coli* O157:H7. Positive burger: the number of burger samples testing positive for *E. coli* O157:H7. Total positive: the combined number of positive samples (minced and burger) for each location. The chi-square test showed no significant difference between the different areas (*p* = 0.838).

**Table 5:** Antimicrobial susceptibility pattern of *E. coli* O157:H7 isolates (*n* = 23).

**Antibacterial agent**	**Resistant**	**Intermediate**	**Sensitive**
Piperacillin/tazobactam	—	1	22
Piperacillin	3 (13%)	9	11
Ampicillin	22 (96%)	—	1
Amoxicillin/clavulanic	22 (96%)	1	—
Ceftriaxone	2 (9%)	3	18
Cefepime	—	1	22
Ceftazidime	1 (4%)	1	21
Ciprofloxacin	1 (4%)	6	16
Norfloxacin	1 (4%)	1	21
Imipenem	3 (13%)	6	14
Meropenem	—	2	21
Aztreonam	—	2	21
Gentamicin	5 (22%)	3	15
Chloramphenicol	10 (43%)	7	6
Trimethoprim/sulfa	14 (61%)	—	9

**Table 6:** Colistin resistance profile of *E. coli* O157:H7 isolates.

	**Sample ** **n** **°**	**Area**	**MIC of colistin (*μ*g/mL)**	***mcr-2***	***mcr-3***
Minced beef samples	1	Chiyah	64	−	−
	2	Chiyah	2	−	−
	4	Haret Hrek	4	−	+
	5	Haret Hrek	32	−	+
	6	Haret Hrek	32	−	+
	8	Basta	2	−	−
	12	Borj Hammoud	8	−	−
	15	Borj Hammoud	≥ 128	−	+
	16	Borj al Barajneh	≥ 128	−	+
	17	Borj al Barajneh	32	+	−
	18	Jnah	64	−	+
	20	Jnah	≥ 128	+	+

Burger samples	21	Haret Hrek	32	−	+
	22	Haret Hrek	32	−	−
	23	Jnah	128	−	−
	24	Basta	128	+	−
	25	Borj Hammoud	128	−	+
	26	Borj Hammoud	128	−	−
	27	Borj al Barajneh	1	−	+
	28	Borj al Barajneh	128	−	−
	29	Borj al Barajneh	128	−	−
	31	Basta	128	+	−
	32	Chiyah	32	−	−

*Note:* ANOVA test/Kruskal–Wallis test revealed no statistical difference in MIC between different areas nor between minced beef and burger (*p* = 0.091 and 0.162, respectively).

## Data Availability

The data that supports the findings of this study are available from the corresponding author upon reasonable request.
